# The Construction and Performance Evaluation of a Risk Prediction Model for Nonalcoholic Steatohepatitis Based on Serological Markers

**DOI:** 10.1155/grp/2580446

**Published:** 2025-10-14

**Authors:** Dongbo Huang, Wanqin Zhang, Ying Fang, Taotao Liu, Da Zhou

**Affiliations:** ^1^Department of Gastroenterology and Hepatology, Zhongshan Hospital of Fudan University, Shanghai, China; ^2^Department of Gastroenterology, Shanghai Xuhui Center Hospital, Shanghai, China

**Keywords:** nomogram, nonalcoholic steatohepatitis, prediction model

## Abstract

**Background and Aims:**

To develop a noninvasive clinical diagnostic model based on serological markers for nonalcoholic steatohepatitis (NASH) and to verify its predictive efficacy.

**Methods:**

A total of 82 biopsy-proven patients with nonalcoholic fatty liver disease (NAFLD) were included in the study. Patients were classified into nonalcoholic fatty liver (NAFL) and NASH groups based on the results of liver biopsies. The study utilized the LASSO regression model for variable selection, followed by logistic regression analysis to create a prediction model. A nomogram was then developed to illustrate this model. To validate the model, bootstrapping was applied for internal validation, and the accuracy, consistency, and clinical utility of the prediction model were evaluated.

**Results:**

The NASH group had significantly higher levels of red blood cell count, lactate dehydrogenase (LDH), aspartate aminotransferase (AST), and alanine aminotransferase (ALT), while levels of high-density lipoprotein (HDL) cholesterol were significantly lower in the NASH group (*p* < 0.05). Logistic regression analysis indicated that AST and ceruloplasmin were independent risk factors associated with NASH. A nomogram based on serological markers, including ceruloplasmin, HDL, AST, red blood cell count, thyroid-stimulating hormone (TSH), and total bile acid (TBA), was established to predict NASH with excellent discrimination (AUROC 0.813).

**Conclusions:**

AST and ceruloplasmin are independent risk factors associated with NASH. The CHART2 prediction model based on serological markers demonstrates good accuracy, consistency, and clinical utility. The model could serve as a noninvasive approach to identifying patients with NASH, which might reduce the need for liver biopsy.

## 1. Introduction

Nonalcoholic steatohepatitis (NASH) is a significant inflammatory subtype of nonalcoholic fatty liver disease (NAFLD). Compared to patients with nonalcoholic fatty liver (NAFL), those with NASH exhibit faster disease progression and worse prognoses. Over 20% of NASH patients eventually develop cirrhosis. Studies show that NASH patients are more likely to experience metabolic abnormalities, including obesity, Type 2 diabetes, hyperlipidemia, and hypertension, when compared to the general NAFLD population. Moreover, NASH patients have a higher incidence of advanced liver fibrosis and are at a significantly increased risk of developing hepatocellular carcinoma (HCC) compared to the broader NAFLD population [1]. Research suggests that NASH has become the leading indication for liver transplantation in American women and is projected to surpass alcoholic liver disease as the primary cause for liver transplantation in patients with end-stage liver disease [2]. Additionally, NASH patients are more prone to extrahepatic complications such as atherosclerotic cardiovascular disease, cerebrovascular disease, renal disease, and certain malignancies. Due to both intrahepatic and extrahepatic complications, the survival rate of NASH patients is significantly lower than that of the general population. Therefore, early diagnosis, effective prevention, and treatment are essential for improving prognosis and quality of life in NASH patients.

Currently, liver biopsy is the gold standard for diagnosing NAFLD and NASH in clinical settings. However, as an invasive procedure, it is limited by sampling errors and complications, including bleeding, infection, bile leakage, and even death. As a result, there is an urgent need for noninvasive diagnostic methods for NASH. Although alanine aminotransferase (ALT) is frequently used as a serological marker, it has a poor correlation with NASH and cannot be relied upon for diagnosis [2]. Cytokeratin-18 (CK-18) is recognized as one of the most validated blood biomarkers for NASH, yet its clinical accuracy remains insufficient [3]. While some diagnostic models for NASH have been proposed, they have not gained widespread use due to factors such as limited population representativeness and complex calculations.

Thus, the development of universally accepted, reliable noninvasive diagnostic methods for NASH remains a critical unmet need. This study is aimed at establishing a noninvasive clinical diagnostic model for NASH based on serological markers and presenting it using a nomogram.

## 2. Methods

### 2.1. Study Population

This retrospective study included 127 patients who underwent liver biopsy between September 2021 and November 2023. The inclusion criteria were as follows: (1) age between 18 and 75 years, (2) evidence of hepatic steatosis or fatty liver as indicated by ultrasound, and (3) availability of complete data for statistical analysis. The exclusion criteria were as follows: (1) excessive alcohol consumption (> 30 g/day for men or > 20 g/day for women), (2) presence of other liver diseases, such as Wilson's disease, hepatitis B or C infection, or drug-induced liver injury, and (3) liver biopsy showing steatosis in less than 5% of hepatocytes. The study adhered to the guidelines of the Declaration of Helsinki and was approved by the Medical Ethics Committee of Zhongshan Hospital of Fudan University (Approval No. B2020-085R). Informed consent was obtained from all patients prior to liver biopsy.

### 2.2. Data Collection and Laboratory Assessment

Demographic characteristics and medical histories were retrieved from the hospital's electronic medical record system. Venous blood samples were collected on the same day as the liver biopsy, following an overnight fast of at least 8 h. Laboratory assessments for all patients included complete blood count, thyroid function markers, liver and kidney function markers, glucose metabolism markers, lipid metabolism markers, iron and copper metabolism markers, alpha-fetoprotein, C-reactive protein, and other relevant parameters.

### 2.3. Vibration-Controlled Transient Elastography (VCTE) Examination

All enrolled patients underwent a VCTE examination using the FibroScan 502 device (Echosens). Liver stiffness measurements (LSMs) were recorded following the examination.

### 2.4. Histopathology Evaluation

Liver samples were analyzed independently and blinded by two experienced pathologists using the Steatosis-Activity-Fibrosis (SAF) scoring algorithm. The diagnosis of NAFLD was based on the following criteria: (1) the presence of steatosis in at least 5% of hepatocytes on liver biopsy and (2) exclusion of other liver diseases. NASH was defined by a histological score of at least 1 point in each of the following features: hepatic steatosis, hepatocellular ballooning, and lobular inflammation [2].

### 2.5. Statistical Analysis

Statistical analysis was performed using the SPSS 27.0 software (SPSS Inc., Chicago, Illinois) and R statistical software (Foundation for Statistical Computing, Vienna, Austria). Categorical variables were presented as frequencies and percentages and analyzed using the chi-square test or Fisher's exact test. The normality of continuous variables was assessed using the Shapiro–Wilk test. Data following normal distribution were expressed as $\text{mean}\pm \text{standard deviation }\left(\bar{X}\pm \text{SD}\right)$, while nonnormally distributed data were presented as median and interquartile range. Intergroup comparisons were conducted using the *t*-test or Mann–Whitney *U* test when appropriate. All statistical analyses were two-tailed, with a 95% confidence interval (CI), and statistical significance was defined as *p* < 0.05. LASSO regression was applied for variable selection. Multivariable logistic regression was used to analyze the risk factors associated with NASH and to develop a prediction model for distinguishing NASH from NAFLD. The nomogram was constructed to estimate the probability of NASH. The diagnostic performance of the model was evaluated using receiver operating characteristic (ROC) curve analysis.

## 3. Results

### 3.1. General Characteristics

**Figure 1:**
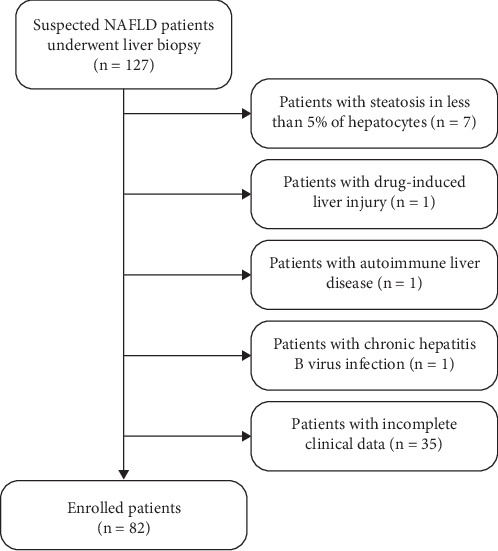
Flowchart of the studied patients.

A total of 82 adult patients with biopsy-proven NAFLD were enrolled in this study. Of these, 60 cases were classified into the NASH group and 22 into the NAFL group, based on liver biopsy results. The patient enrollment flowchart is shown in Figure 1, and the general characteristics of the study population are summarized in Table 1. Compared to patients in the NAFL group, those in the NASH group had significantly higher levels of red blood cell counts, ALT, AST, and LDH, while HDL levels were significantly lower (*p* < 0.05). No significant differences were observed in other parameters, including LSMs (*p* > 0.05 for all comparisons).

### 3.2. Construction of the CHART2 Model for Discriminating NASH

**Figure 2:**
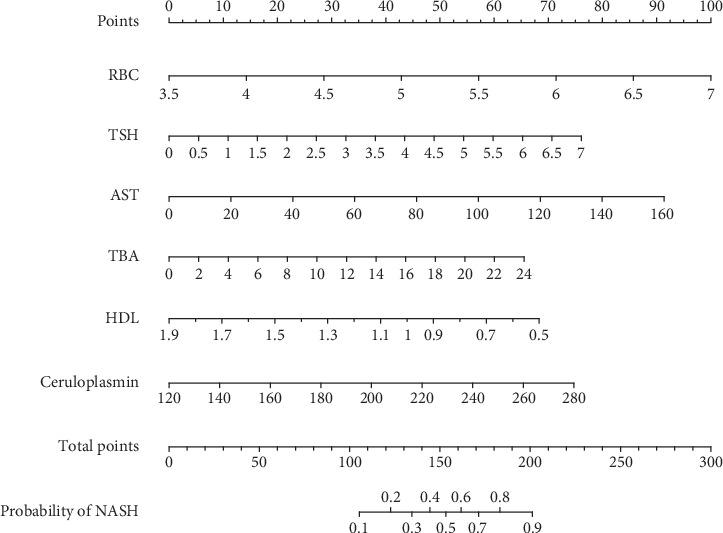
The nomogram of the predictive model for discriminating NASH.

LASSO regression analysis identified eight potential predictive factors: red blood cell count, total bile acid (TBA), ceruloplasmin, thyroid-stimulating hormone (TSH), ALT, AST, HDL, and uric acid (UA). These eight variables were then included in a binary logistic regression. The analysis revealed that six variables—red blood cell count, AST, TSH, HDL, TBA, and ceruloplasmin—were incorporated into the regression model. Among them, AST (OR: 1.027, 95% CI: 1.004–1.055, *p* = 0.035) and ceruloplasmin (OR: 1.022, 95% CI: 1.002–1.044, *p* = 0.038) were independent risk predictors for NASH (Table 2). The regression formula for predicting a patient's risk of developing NASH was as follows: *P* = *e*^logit (NASH)^/1 + *e*^logit (NASH)^; logit(NASH) = −9.941 + 1.312 × red blood cell count (10^12^/L) + 0.026 × AST (U/L) − 2.239 × HDL (mmol/L) + 0.499 × TSH (*μ*IU/mL) + 0.125 × TBA (*μ*mol/L) + 0.021 × ceruloplasmin (mg/L). Subsequently, these six variables were further analyzed using R statistical software to construct a personalized nomogram for NASH risk (see Figure 2).

### 3.3. Performance of the CHART2 Model in Discriminating NASH From NAFLD Patients

#### 3.3.1. Discriminative Ability of the Model

**Figure 3:**
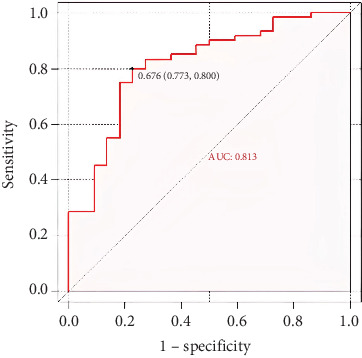
The performance of the predictive model in discriminating NASH.

To assess the discriminative ability of the CHART2 model, a ROC curve analysis was conducted. The area under the ROC curve (AUROC) for the model was 0.813 (95% CI: 0.706–0.920), demonstrating a high level of accuracy in distinguishing NASH from NAFLD patients (see Figure 3).

#### 3.3.2. Calibration of the Model

**Figure 4:**
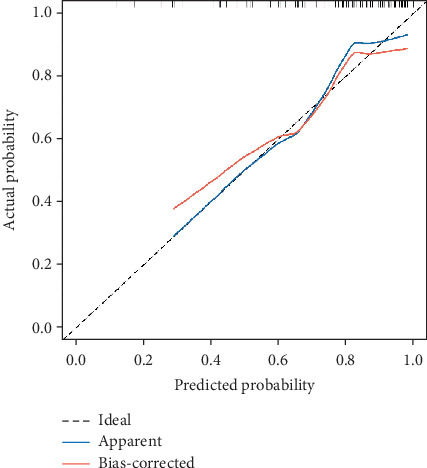
The calibration curves for the nomogram.

The calibration of the CHART2 model was assessed using the Hosmer–Lemeshow test, which yielded a *p* value of 0.627, indicating a strong agreement between the model's predictions and actual observations. Additionally, bootstrapping was performed for internal validation, and a calibration curve was plotted. The calibration curve closely aligned with the reference line, further confirming the model's accuracy and consistency (see Figure 4).

#### 3.3.3. Clinical Utility of the Model

**Figure 5:**
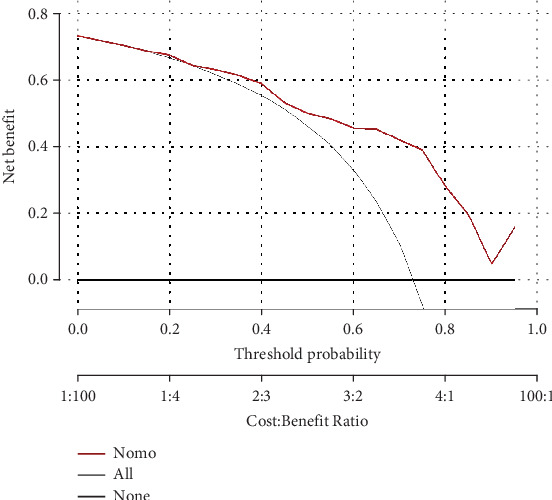
Clinical decision curve of the predictive model for discriminating NASH.

The clinical utility of the CHART2 model was evaluated through a decision curve analysis (DCA). The results showed that when the risk threshold probability exceeds 25%, the net benefit of using the CHART2 model surpasses that of the two extreme curves. This indicates that within this risk threshold range, the model provides significant clinical benefit in identifying NASH patients. Furthermore, the wide range of applicable risk thresholds underscores the model's robust clinical utility (see Figure 5).

## 4. Discussion

In this study, we conducted a retrospective analysis of NAFLD cases diagnosed via liver biopsy to identify potential predictive factors for NASH and developed the CHART2 model for distinguishing NASH from NAFLD. We utilized LASSO regression to select optimal predictive variables and subsequently incorporated six variables—red blood cell count, AST, TSH, HDL, TBA, and ceruloplasmin—into the predictive model, which was presented as a nomogram. The model demonstrated an AUROC of 0.813, and both the calibration curve and the Hosmer–Lemeshow test indicated strong predictive consistency. DCA further confirmed the model's significant clinical utility. The CHART2 model's advantage lies in its use of readily available clinical biochemical markers and straightforward calculation methods.

NASH has a significantly lower survival rate compared to the general population, and its rising prevalence poses a substantial burden on public health and socioeconomic systems. Early and noninvasive diagnosis of NASH is crucial for improving patient prognosis and quality of life. Although liver biopsy remains the gold standard for diagnosing NASH, its invasive nature limits widespread clinical application. Consequently, there is a pressing need for noninvasive diagnostic methods. Several noninvasive models, including those based on serological biomarkers, imaging indicators, or algorithms, have been developed. However, many of these models face challenges such as limited applicability or lack of extensive validation. For instance, the Gholam model, which includes serum AST levels and the presence of Type 2 diabetes, has an AUROC of 0.82 but is limited by its study population, which was severely obese (BMI: 55 ± 12 kg/m^2^) and predominantly female [4]. Similarly, the model by Palekar et al., incorporating variables like age, gender, AST, BMI, AST/ALT ratio, and serum hyaluronic acid, has an AUROC of 0.76, with lower sensitivity and specificity [5]. The NashTest scoring model, developed by Poynard et al., with an AUROC of 0.79, has high specificity but low sensitivity and is complex due to its 13 variables and inclusion of less commonly tested markers [6]. In contrast, the CHART2 model, with an AUROC of 0.813 and sensitivity and specificity of 80% and 77.3%, respectively, uses more accessible biomarkers and simpler calculations, making it more suitable for routine clinical use. Wu et al.'s acNASH index model, with an AUROC of 0.805–0.818, identifies NASH through serum creatinine and AST levels but has not performed well in other studies [7]. Thus, our predictive model offers a novel, noninvasive approach for identifying NASH patients, potentially reducing reliance on liver biopsies.

Our study identified elevated AST as an independent risk factor for NASH, consistent with findings from Younossi et al. and Feldstein et al. [8, 9]. AST, a mitochondrial enzyme released during hepatocyte damage, may reflect mitochondrial injury in NASH patients. Elevated serum AST levels are commonly included in NASH predictive models, emphasizing its predictive significance. However, the optimal timing for using serum AST levels in clinical practice must be established, as it can be influenced by hepatoprotective agents.

Additionally, ceruloplasmin emerged as an independent predictor for NASH. As a copper-containing protein secreted by the liver, ceruloplasmin plays a key role in iron homeostasis [10]. Studies have found that serum ceruloplasmin levels are negatively correlated with liver fat content [11]. There is also evidence that ceruloplasmin with enzymatic activity can increase iron efflux from the liver [12], leading some researchers to suggest that reduced ceruloplasmin may result in iron deposition in the liver, which is closely associated with the development of NAFLD/NASH [13]. Iron accumulated in hepatocytes can increase the production of reactive oxygen species through the Fenton reaction, thereby inducing inflammation, fibrosis, and apoptosis. Some studies have found that lower serum ceruloplasmin levels are significantly associated with NASH and can be used to predict the onset of NASH [14]. Conversely, other studies have reported increased levels of serum ceruloplasmin in patients with diabetes, metabolic syndrome, and NAFLD [15–17]. Given these ongoing controversies, we systematically evaluated the impact of ceruloplasmin exclusion in our modeling approach. Our analyses revealed that the exclusion of ceruloplasmin resulted in a complete failure to derive a valid logistic regression equation. Consequently, the predictive model could not be properly constructed. Therefore, we ultimately included this variable in our predictive model. The regression equation generated in this study when constructing the predictive model appears to support a higher ceruloplasmin level (OR 1.022, 95% CI: 1.002–1.044) being associated with NASH. A possible reason is that ceruloplasmin, as an acute-phase reactant protein, may have its serum concentration increased during inflammation, infection, and trauma due to cytokine-mediated hepatocyte gene transcription [18]. To date, the role of ceruloplasmin in the pathogenesis of NASH remains incompletely elucidated, and further research is needed.

This study has limitations, including a single-center design and a relatively small sample size, which restrict generalizability and necessitate confirmation through larger, external validation cohorts. Additionally, the retrospective nature of the study and the selection of clinical tests may have introduced data bias and omitted valuable predictive variables.

In conclusion, the nomogram based on serological markers could be used as a valuable tool for noninvasive risk prediction of NASH, facilitating the differentiation of NASH from NAFLD in clinical practice.

## Figures and Tables

**Table 1:** Demographic and clinical characteristics of enrolled patients in the NASH and NAFL groups.

**Characters**	**NASH (** **n** = 60**)**	**NAFL (** **n** = 22**)**	**p** ** value**
Age (years)	42.0 [34.0, 56.5]	50.5 [35.8, 65.0]	0.203
Gender (male %)	37 (61.7%)	10 (45.5%)	0.288
Height (cm)	168 ± 9.07	164 ± 8.03	0.071
Weight (kg)	80.1 ± 16.4	74.4 ± 11.9	0.089
BMI (kg/m^2^)	28.1 ± 3.92	27.4 ± 3.21	0.442
IFG/diabetes	13 (21.7%)	9 (40.9%)	0.144
AFP (ng/mL)	2.60 [2.00, 3.32]	2.25 [1.83, 4.10]	0.683
RBC (10^12^/L)	4.84 ± 0.54	4.53 ± 0.44	0.012^∗^
Hemoglobin (g/L)	144 ± 14.7	138 ± 14.7	0.135
WBC (10^9^/L)	6.30 [5.46, 7.46]	5.66 [4.94, 6.58]	0.101
Platelets (10^9^/L)	225 ± 62.5	233 ± 50.8	0.556
fT3 (pmol/L)	4.95 [4.40, 5.53]	4.75 [4.43, 5.20]	0.280
fT4 (pmol/L)	16.0 (2.38)	16.5 (2.27)	0.344
TSH (*μ*IU/mL)	1.84 [1.46, 2.83]	1.55 [1.22, 1.90]	0.081
Total bilirubin (*μ*mol/L)	12.2 [9.60, 16.3]	12.1 [10.0, 15.0]	0.773
Direct bilirubin (*μ*mol/L)	3.30 [2.40, 4.03]	3.25 [2.08, 4.27]	0.929
Albumin (g/L)	46.1 ± 3.27	44.7 ± 2.86	0.078
ALT (U/L)	92.5 [61.0, 139]	58.0 [30.0, 100]	0.014^∗^
AST (U/L)	48.5 [37.8, 67.2]	33.0 [22.5, 48.8]	0.004^∗^
AKP (U/L)	75.0 [65.8, 93.0]	68.5 [60.2, 76.8]	0.146
GGT (U/L)	69.0 [47.2, 85.2]	54.5 [33.5, 83.5]	0.211
TBA (*μ*mol/L)	5.75 [3.27, 8.10]	4.75 [2.75, 5.60]	0.136
LDH (U/L)	190 [166, 217]	166 [151, 186]	0.012^∗^
Prealbumin (mg/L)	242 [213, 277]	244 [214, 275]	0.672
Creatinine (*μ*mol/L)	73.1 ± 16.1	71.9 ± 18.3	0.788
BUN (mmol/L)	4.60 [4.00, 5.62]	4.70 [4.23, 5.57]	0.753
Uric acid (mmol/L)	400 [347, 469]	381 [306, 438]	0.179
Glucose (mmol/L)	5.20 [4.80, 5.90]	5.20 [4.80, 5.97]	0.863
Total cholesterol (mmol/L)	4.82 [4.36, 5.53]	4.72 [3.98, 5.49]	0.626
Triglycerides (mg/dL)	152 [117, 196]	150 [102, 176]	0.298
HDL (mmol/L)	1.04 ± 0.21	1.18 ± 0.28	0.040^∗^
Non-HDL cholesterol (mmol/L)	3.86 [3.35, 4.41]	3.76 [2.96, 4.55]	0.426
LDL (mmol/L)	2.88 [2.55, 3.51]	2.90 [2.37, 3.46]	0.842
Apolipoprotein A–I (g/L)	1.34 ± 0.20	1.44 ± 0.33	0.199
Apolipoprotein B (g/L)	0.94 [0.78, 1.06]	0.92 [0.72, 1.08]	0.513
Ceruloplasmin (mg/L)	202 ± 28.6	194 ± 31.5	0.349
HOMA-IR	3.88 [2.46, 5.41]	3.10 [2.60, 4.47]	0.237
LSM (kPa)	10.3 [8.45, 13.2]	11.2 [8.05, 13.0]	0.601

Abbreviations: AFP, alpha-fetoprotein; AKP, alkaline phosphatase; ALT, alanine transferase; AST, aspartate transferase; BMI, body mass index; BUN, blood urea nitrogen; fT3, free triiodothyronine; fT4, free thyroxine; GGT, gamma- glutamyl transferase; HDL, high-density lipoprotein; HOMA-IR, homeostasis model assessment of insulin resistance; IFG, impaired fasting glucose; LDL, low-density lipoprotein; LSM, liver stiffness measurement; RBC, red blood cell; TBA, total bile acid; TSH, thyroid-stimulating hormone; WBC, white blood cell.

⁣^∗^*p* value < 0.05.

**Table 2:** Multivariate analysis of risk factors for NASH.

**Parameters**	**β** ** value**	**OR (95% CI)**	**p** ** value**
Intercept	−9.941	0 (0–0.427)	0.045
RBC	1.312	3.712 (0.989–17.47)	0.07
AST	0.026	1.027 (1.004–1.055)	0.035^∗^
HDL	−2.239	0.107 (0.006–1.455)	0.103
TSH	0.499	1.647 (0.981–3.11)	0.085
TBA	0.125	1.134 (0.986–1.356)	0.12
Ceruloplasmin	0.021	1.022 (1.002–1.044)	0.038^∗^

Abbreviations: AST, aspartate transferase; HDL, high-density lipoprotein; RBC, red blood cell; TBA, total bile acid; TSH, thyroid-stimulating hormone.

⁣^∗^*p* value < 0.05.

## Data Availability

The data that support the findings of this study are available on request from the corresponding authors. The data are not publicly available due to privacy or ethical restrictions.
